# Bis(μ-pyridine-2,5-dicarboxyl­ato)-κ^3^ *N*,*O* ^2^:*O* ^5^;κ^3^ *O* ^5^:*N*,*O* ^2^-bis­[aqua­(2,2′-bipyridine-κ^2^ *N*,*N*′)cobalt(II)] dihydrate

**DOI:** 10.1107/S1600536811003059

**Published:** 2011-01-29

**Authors:** Shie Fu Lush

**Affiliations:** a Department of Genernal Eduction Center, Yuanpei University, HsinChu 30015, Taiwan

## Abstract

In the centrosymmetric title compound, [Co_2_(C_7_H_3_NO_4_)_2_(C_10_H_8_N_2_)_2_(H_2_O)_2_]·2H_2_O, the two Co^II^ cations are bridged by pairs of pyridine-2,5-dicarboxyl­ate anions across an inversion center. Besides two pyridine-2,5-dicarboxyl­ate anions, one bidentate 2,2′-bipyridine and one water mol­ecule coordinate to the Co cation, completing a distorted octa­hedral coordination geometry. Within the dinuclear mol­ecule, π–π stacking occurs between parallel pyridine rings with centroid–centroid distances of 3.802 (2) Å. The crystal structure contains extensive O—H⋯O and weak C—H⋯O hydrogen bonds and C—H⋯π inter­actions.

## Related literature

For multi-dentate coordination modes of the pyridine-3,5-dicarboxyl­ate anion, see: Gao *et al.* (2005 ▶). For related structures, see: Aghabozorg *et al.* (2007 ▶); Lu *et al.* (2006 ▶); Xu *et al.* (2004 ▶).
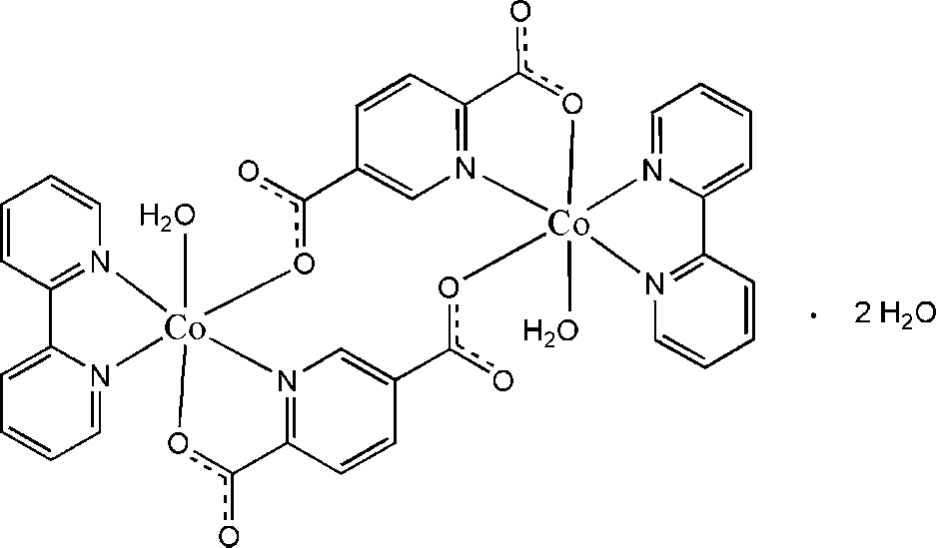


## Experimental

### 

#### Crystal data

[Co_2_(C_7_H_3_NO_4_)_2_(C_10_H_8_N_2_)_2_(H_2_O)_2_]·2H_2_O
*M* *_r_* = 832.50
Triclinic, 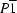

*a* = 7.2731 (11) Å
*b* = 9.7123 (15) Å
*c* = 12.2887 (19) Å
α = 98.447 (3)°
β = 103.283 (3)°
γ = 91.564 (3)°
*V* = 834.0 (2) Å^3^
*Z* = 1
Mo *K*α radiation
μ = 1.07 mm^−1^
*T* = 294 K
0.30 × 0.20 × 0.20 mm

#### Data collection

Bruker SMART CCD area-detector diffractometer
Absorption correction: multi-scan (*SADABS*; Bruker, 2001 ▶) *T*_min_ = 0.928, *T*_max_ = 0.971
4799 measured reflections
2903 independent reflections
2557 reflections with *I* > 2σ(*I*)
*R*_int_ = 0.031

#### Refinement

*R*[*F* ^2^ > 2σ(*F* ^2^)] = 0.056
*wR*(*F* ^2^) = 0.145
*S* = 1.14
2903 reflections
244 parameters
H-atom parameters constrained
Δρ_max_ = 1.58 e Å^−3^
Δρ_min_ = −0.37 e Å^−3^

### 

Data collection: *SMART* (Bruker, 2007 ▶); cell refinement: *SAINT* (Bruker, 2007 ▶); data reduction: *SAINT*; program(s) used to solve structure: *SHELXS97* (Sheldrick, 2008 ▶); program(s) used to refine structure: *SHELXL97* (Sheldrick, 2008 ▶); molecular graphics: *ORTEP-3 for Windows* (Farrugia, 1997 ▶); software used to prepare material for publication: *PLATON* (Spek, 2009 ▶).

## Supplementary Material

Crystal structure: contains datablocks global, I. DOI: 10.1107/S1600536811003059/xu5145sup1.cif

Structure factors: contains datablocks I. DOI: 10.1107/S1600536811003059/xu5145Isup2.hkl

Additional supplementary materials:  crystallographic information; 3D view; checkCIF report

## Figures and Tables

**Table 1:** Selected bond lengths (Å)

Co1—O1	2.059 (3)
Co1—O2	2.056 (3)
Co1—O5^i^	2.089 (3)
Co1—N1	2.126 (3)
Co1—N2	2.155 (3)
Co1—N3	2.204 (3)

Symmetry code: (i) 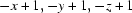.

**Table 2:** Hydrogen-bond geometry (Å, °) *Cg* is the centroid of the *N*-pyridine ring.

*D*—H⋯*A*	*D*—H	H⋯*A*	*D*⋯*A*	*D*—H⋯*A*
O1—H1*A*⋯O3^ii^	0.84	1.82	2.629 (4)	159
O1—H1*B*⋯O6	0.88	1.94	2.755 (5)	154
O6—H6*A*⋯O4^iii^	0.85	2.40	3.232 (5)	166
O6—H6*B*⋯O4^iv^	0.83	2.01	2.829 (5)	170
C4—H4*A*⋯O2^v^	0.93	2.52	3.202 (5)	131
C7—H7*A*⋯O6^vi^	0.93	2.57	3.332 (6)	140
C8—H8*A*⋯O4^vii^	0.93	2.55	3.461 (6)	169
C9—H9*A*⋯O3^viii^	0.93	2.59	3.330 (6)	137
C12—H12*A*⋯O4^ix^	0.93	2.44	3.317 (5)	158
C15—H15*A*⋯O3^ii^	0.93	2.36	3.249 (5)	160
C2—H2*A*⋯*Cg*^iv^	0.93	2.72	3.547 (5)	150

Symmetry codes: (ii) 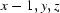; (iii) 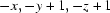; (iv) 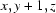; (v) 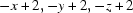; (vi) 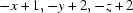; (vii) 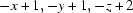; (viii) 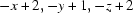; (ix) 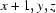.

**Fig. 1.**
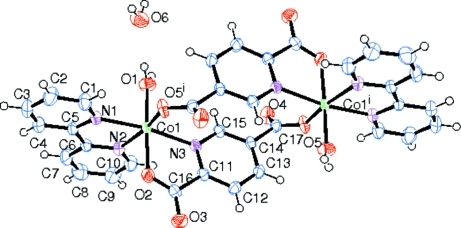
View of the title compound with the atom numbering scheme. Displacement ellipsoids for non-H atoms are drawn at the 50% probability level. All H atoms have been omitted for clarity [symmetry code: (i) 1 - x, 1 - y, 1 - z].

**Fig. 2.**
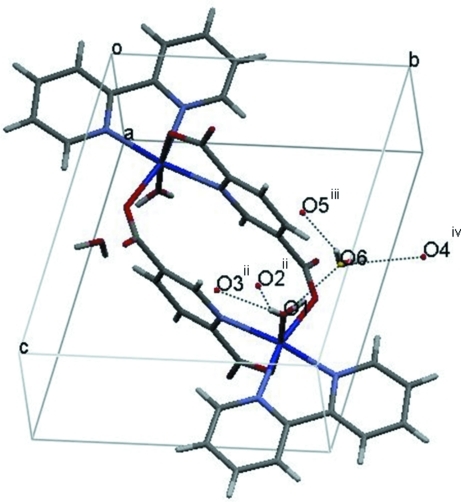
The molecular packing for the title compound. Hydrogen bonds are shown as dashed lines.

## supplementary crystallographic information

### Comment

2,5-PydH_2_ can be easily deprotonated to get a O-donors to N-donor multidentate anion (pyd^2-^), enabling the ligand coordinates to two or more metal ions in a briding mode and chelating mode (Gao *et al.*, 2005). The complexation of metal ions using the deprotonated conjugate base of 2,5-pydH_2_ as a ligand has been reported in the literature (Aghabozorg *et al.*, 2007; Lu *et al.*, 2006; Xu *et al.*, 2004).

The symmetric unit of the title compound contain two Co^II^ cations, two pyd anions, two bpy molecules and two coordinated water molecules. Each Co^II^ ion is surrounded by one bpy ligand, two pyd anions and one coordinated water molecule, to give a distorted octahedral geometry. Two pyd anions bridges two Co^II^ ions to form a centrosymmetric dimeric complex (Table 1 and Fig. 1).

The crystal structure contains an extensive network of classical O—H···O and weak C—H···O hydrogen bonds (full details and symmetry codes are given in Table 2 and Fig. 2). C—H ···π(full detail and symmetry code is given in Table 2) and π-π stacking are present in the crystal structure, the shortest centroids distance between parallel pyridine rings is 3.8023 (17) Å [C*g*5^i^···C*g*5 (N3/C11—C15)] [symmetry code: 1 - *x*, 1 - *y*, 1 - *z*].

### Experimental

An aqueous solution (5 ml) of bpy (0.0312 g, 0.20 mmol) was mixed with aqueous solution (5 ml) containing Co(NO_3_)_2_.6H_2_O (0.0450 g, 0.20 mmol) and 2,5-pydH_2_ (0.0346 g, 0.2 mmol). The mxiture was put in a 23-ml Teflon liner reactor and heated at 418 K in oven for 48 h. The resulting solution was slowly cooled to room temperature. The orange transparent single crystals of the title complex were obtained in 35.5% yield (based on Co).

### Refinement

Water H atoms were placed in chemical sensible positions and refined in a riding model. Other H atoms were positioned geometrically with C—H = 0.93 Å and refined using a riding model. *U*_iso_(H) = 1.2*U*_eq_(O,*C*).

### Crystal data

**Table:**

[Co_2_(C_7_H_3_NO_4_)_2_(C_10_H_8_N_2_)_2_(H_2_O)_2_]·2H_2_O	*Z* = 1
*M**_r_* = 832.50	*F*(000) = 426
Triclinic, *P*1	*D*_x_ = 1.658 Mg m^−^^3^
Hall symbol: -P 1	Mo *K*α radiation, λ = 0.71073 Å
*a* = 7.2731 (11) Å	Cell parameters from 2565 reflections
*b* = 9.7123 (15) Å	θ = 2.5–25.0°
*c* = 12.2887 (19) Å	µ = 1.07 mm^−^^1^
α = 98.447 (3)°	*T* = 294 K
β = 103.283 (3)°	Columnar, orange
γ = 91.564 (3)°	0.30 × 0.20 × 0.20 mm
*V* = 834.0 (2) Å^3^	

### Data collection

**Table:**

Bruker SMART CCD area-detector diffractometer	2903 independent reflections
Radiation source: fine-focus sealed tube	2557 reflections with *I* > 2σ(*I*)
graphite	*R*_int_ = 0.031
Detector resolution: 9 pixels mm^-1^	θ_max_ = 25.0°, θ_min_ = 1.7°
φ and ω scans	*h* = −8→8
Absorption correction: multi-scan (*SADABS*; Bruker, 2001)	*k* = −11→11
*T*_min_ = 0.928, *T*_max_ = 0.971	*l* = −14→14
4799 measured reflections	

### Refinement

**Table:**

Refinement on *F*^2^	Primary atom site location: structure-invariant direct methods
Least-squares matrix: full	Secondary atom site location: difference Fourier map
*R*[*F*^2^ > 2σ(*F*^2^)] = 0.056	Hydrogen site location: inferred from neighbouring sites
*wR*(*F*^2^) = 0.145	H-atom parameters constrained
*S* = 1.14	*w* = 1/[σ^2^(*F*_o_^2^) + (0.0875*P*)^2^ + 0.435*P*] where *P* = (*F*_o_^2^ + 2*F*_c_^2^)/3
2903 reflections	(Δ/σ)_max_ < 0.001
244 parameters	Δρ_max_ = 1.58 e Å^−^^3^
0 restraints	Δρ_min_ = −0.37 e Å^−^^3^

### Special details

**Table:**

Geometry. All e.s.d.'s (except the e.s.d. in the dihedral angle between two l.s. planes) are estimated using the full covariance matrix. The cell e.s.d.'s are taken into account individually in the estimation of e.s.d.'s in distances, angles and torsion angles; correlations between e.s.d.'s in cell parameters are only used when they are defined by crystal symmetry. An approximate (isotropic) treatment of cell e.s.d.'s is used for estimating e.s.d.'s involving l.s. planes.
Refinement. Refinement of *F*^2^ against ALL reflections. The weighted *R*-factor *wR* and goodness of fit *S* are based on *F*^2^, conventional *R*-factors *R* are based on *F*, with *F* set to zero for negative *F*^2^. The threshold expression of *F*^2^ > σ(*F*^2^) is used only for calculating *R*-factors(gt) *etc*. and is not relevant to the choice of reflections for refinement. *R*-factors based on *F*^2^ are statistically about twice as large as those based on *F*, and *R*- factors based on ALL data will be even larger.

### Fractional atomic coordinates and isotropic or equivalent isotropic displacement parameters (Å^2^)

**Table:**

	*x*	*y*	*z*	*U*_iso_*/*U*_eq_	
Co1	0.66185 (7)	0.76879 (5)	0.74280 (4)	0.0247 (2)	
O1	0.3772 (4)	0.7870 (3)	0.7328 (2)	0.0354 (7)	
H1A	0.2956	0.7189	0.7125	0.042*	
H1B	0.3279	0.8561	0.6992	0.042*	
O2	0.9431 (4)	0.7317 (3)	0.7591 (2)	0.0364 (7)	
O3	1.1448 (4)	0.5714 (3)	0.7242 (3)	0.0435 (8)	
O4	0.2217 (4)	0.2111 (3)	0.5635 (3)	0.0432 (8)	
O5	0.3331 (4)	0.1622 (3)	0.4098 (2)	0.0381 (7)	
O6	0.1195 (5)	0.9586 (4)	0.6300 (3)	0.0535 (9)	
H6A	0.0183	0.9165	0.5883	0.064*	
H6B	0.1367	1.0315	0.6048	0.064*	
N1	0.7131 (5)	0.9779 (3)	0.8284 (3)	0.0295 (7)	
N2	0.6948 (5)	0.7464 (3)	0.9180 (3)	0.0305 (8)	
N3	0.6456 (4)	0.5456 (3)	0.6717 (3)	0.0254 (7)	
C1	0.7223 (7)	1.0907 (5)	0.7791 (4)	0.0396 (10)	
H1C	0.7019	1.0789	0.7007	0.047*	
C2	0.7609 (7)	1.2242 (5)	0.8393 (4)	0.0466 (12)	
H2A	0.7656	1.3007	0.8024	0.056*	
C3	0.7921 (7)	1.2406 (5)	0.9552 (4)	0.0464 (12)	
H3A	0.8213	1.3288	0.9982	0.056*	
C4	0.7800 (6)	1.1259 (5)	1.0069 (4)	0.0378 (10)	
H4A	0.7985	1.1362	1.0852	0.045*	
C5	0.7403 (5)	0.9954 (4)	0.9422 (3)	0.0282 (9)	
C6	0.7270 (5)	0.8659 (4)	0.9919 (3)	0.0289 (9)	
C7	0.7501 (7)	0.8667 (5)	1.1072 (4)	0.0415 (11)	
H7A	0.7716	0.9507	1.1569	0.050*	
C8	0.7409 (7)	0.7430 (6)	1.1474 (4)	0.0481 (12)	
H8A	0.7565	0.7422	1.2245	0.058*	
C9	0.7083 (7)	0.6200 (5)	1.0724 (4)	0.0465 (12)	
H9A	0.7011	0.5346	1.0974	0.056*	
C10	0.6866 (7)	0.6277 (5)	0.9595 (4)	0.0391 (10)	
H10A	0.6648	0.5446	0.9087	0.047*	
C11	0.8176 (5)	0.5074 (4)	0.6603 (3)	0.0262 (8)	
C12	0.8460 (6)	0.3818 (4)	0.5996 (4)	0.0325 (9)	
H12A	0.9669	0.3591	0.5935	0.039*	
C13	0.6905 (6)	0.2898 (4)	0.5480 (3)	0.0310 (9)	
H13A	0.7052	0.2058	0.5047	0.037*	
C14	0.5124 (5)	0.3253 (4)	0.5620 (3)	0.0247 (8)	
C15	0.4972 (5)	0.4536 (4)	0.6248 (3)	0.0248 (8)	
H15A	0.3785	0.4771	0.6349	0.030*	
C16	0.9822 (5)	0.6112 (4)	0.7197 (3)	0.0286 (9)	
C17	0.3412 (6)	0.2256 (4)	0.5075 (3)	0.0275 (9)	

### Atomic displacement parameters (Å^2^)

**Table:**

	*U*^11^	*U*^22^	*U*^33^	*U*^12^	*U*^13^	*U*^23^
Co1	0.0275 (3)	0.0238 (3)	0.0205 (3)	−0.0027 (2)	0.0054 (2)	−0.0029 (2)
O1	0.0326 (15)	0.0318 (16)	0.0404 (18)	−0.0027 (12)	0.0074 (13)	0.0042 (13)
O2	0.0257 (14)	0.0385 (17)	0.0376 (17)	−0.0053 (12)	0.0046 (13)	−0.0119 (14)
O3	0.0263 (15)	0.0384 (17)	0.062 (2)	0.0004 (13)	0.0085 (15)	0.0009 (15)
O4	0.0427 (18)	0.0408 (18)	0.047 (2)	−0.0095 (14)	0.0219 (16)	−0.0048 (15)
O5	0.0526 (19)	0.0330 (16)	0.0231 (15)	−0.0100 (14)	0.0044 (13)	−0.0042 (12)
O6	0.053 (2)	0.0413 (19)	0.059 (2)	−0.0054 (15)	−0.0058 (17)	0.0152 (17)
N1	0.0346 (18)	0.0271 (18)	0.0251 (18)	0.0008 (14)	0.0071 (15)	−0.0010 (14)
N2	0.0353 (18)	0.0274 (18)	0.0269 (18)	0.0000 (14)	0.0065 (15)	−0.0002 (14)
N3	0.0274 (16)	0.0267 (17)	0.0220 (17)	0.0018 (13)	0.0066 (13)	0.0023 (13)
C1	0.055 (3)	0.034 (2)	0.029 (2)	0.000 (2)	0.009 (2)	0.0027 (18)
C2	0.066 (3)	0.026 (2)	0.048 (3)	0.003 (2)	0.014 (2)	0.004 (2)
C3	0.057 (3)	0.030 (2)	0.043 (3)	−0.001 (2)	0.005 (2)	−0.010 (2)
C4	0.045 (3)	0.037 (2)	0.028 (2)	0.0016 (19)	0.006 (2)	−0.0040 (18)
C5	0.0266 (19)	0.029 (2)	0.025 (2)	0.0013 (16)	0.0038 (17)	−0.0039 (16)
C6	0.030 (2)	0.032 (2)	0.022 (2)	0.0037 (16)	0.0038 (17)	−0.0002 (16)
C7	0.055 (3)	0.044 (3)	0.022 (2)	0.005 (2)	0.008 (2)	−0.0024 (19)
C8	0.058 (3)	0.062 (3)	0.027 (2)	0.008 (2)	0.010 (2)	0.013 (2)
C9	0.060 (3)	0.044 (3)	0.040 (3)	0.007 (2)	0.014 (2)	0.017 (2)
C10	0.051 (3)	0.035 (2)	0.031 (2)	0.001 (2)	0.010 (2)	0.0042 (19)
C11	0.0253 (19)	0.029 (2)	0.025 (2)	−0.0011 (16)	0.0063 (16)	0.0057 (16)
C12	0.029 (2)	0.033 (2)	0.035 (2)	0.0046 (17)	0.0110 (18)	0.0000 (18)
C13	0.034 (2)	0.031 (2)	0.027 (2)	0.0032 (17)	0.0090 (18)	−0.0020 (17)
C14	0.032 (2)	0.0254 (19)	0.0160 (18)	0.0002 (16)	0.0051 (16)	0.0036 (15)
C15	0.0251 (19)	0.028 (2)	0.0206 (19)	−0.0007 (15)	0.0057 (16)	0.0023 (15)
C16	0.027 (2)	0.035 (2)	0.022 (2)	−0.0029 (17)	0.0051 (16)	0.0033 (17)
C17	0.032 (2)	0.0219 (19)	0.027 (2)	−0.0009 (16)	0.0059 (17)	0.0020 (16)

### Geometric parameters (Å, °)

**Table:**

Co1—O1	2.059 (3)	C2—H2A	0.9300
Co1—O2	2.056 (3)	C3—C4	1.371 (7)
Co1—O5^i^	2.089 (3)	C3—H3A	0.9300
Co1—N1	2.126 (3)	C4—C5	1.379 (6)
Co1—N2	2.155 (3)	C4—H4A	0.9300
Co1—N3	2.204 (3)	C5—C6	1.487 (6)
O1—H1A	0.8446	C6—C7	1.387 (6)
O1—H1B	0.8808	C7—C8	1.370 (7)
O2—C16	1.265 (5)	C7—H7A	0.9300
O3—C16	1.246 (5)	C8—C9	1.375 (7)
O4—C17	1.243 (5)	C8—H8A	0.9300
O5—C17	1.255 (5)	C9—C10	1.374 (6)
O5—Co1^i^	2.089 (3)	C9—H9A	0.9300
O6—H6A	0.8484	C10—H10A	0.9300
O6—H6B	0.8317	C11—C12	1.380 (6)
N1—C1	1.334 (5)	C11—C16	1.516 (5)
N1—C5	1.351 (5)	C12—C13	1.388 (6)
N2—C10	1.333 (6)	C12—H12A	0.9300
N2—C6	1.343 (5)	C13—C14	1.391 (5)
N3—C15	1.344 (5)	C13—H13A	0.9300
N3—C11	1.347 (5)	C14—C15	1.388 (5)
C1—C2	1.382 (6)	C14—C17	1.515 (5)
C1—H1C	0.9300	C15—H15A	0.9300
C2—C3	1.374 (7)		
			
O2—Co1—O1	174.08 (12)	C5—C4—H4A	120.2
O2—Co1—O5^i^	87.58 (13)	N1—C5—C4	121.3 (4)
O1—Co1—O5^i^	96.94 (12)	N1—C5—C6	115.8 (3)
O2—Co1—N1	95.03 (12)	C4—C5—C6	122.9 (4)
O1—Co1—N1	88.82 (12)	N2—C6—C7	121.6 (4)
O5^i^—Co1—N1	89.97 (12)	N2—C6—C5	115.7 (3)
O2—Co1—N2	88.54 (13)	C7—C6—C5	122.7 (4)
O1—Co1—N2	87.96 (12)	C8—C7—C6	119.5 (4)
O5^i^—Co1—N2	165.79 (12)	C8—C7—H7A	120.2
N1—Co1—N2	76.76 (13)	C6—C7—H7A	120.2
O2—Co1—N3	78.33 (11)	C7—C8—C9	119.3 (4)
O1—Co1—N3	97.44 (12)	C7—C8—H8A	120.3
O5^i^—Co1—N3	94.61 (12)	C9—C8—H8A	120.3
N1—Co1—N3	171.73 (12)	C10—C9—C8	117.8 (4)
N2—Co1—N3	97.98 (12)	C10—C9—H9A	121.1
Co1—O1—H1A	123.8	C8—C9—H9A	121.1
Co1—O1—H1B	115.0	N2—C10—C9	124.2 (4)
H1A—O1—H1B	106.9	N2—C10—H10A	117.9
C16—O2—Co1	117.4 (2)	C9—C10—H10A	117.9
C17—O5—Co1^i^	132.6 (3)	N3—C11—C12	123.1 (4)
H6A—O6—H6B	107.3	N3—C11—C16	115.7 (3)
C1—N1—C5	118.4 (4)	C12—C11—C16	121.2 (3)
C1—N1—Co1	125.5 (3)	C11—C12—C13	118.8 (4)
C5—N1—Co1	116.1 (3)	C11—C12—H12A	120.6
C10—N2—C6	117.5 (4)	C13—C12—H12A	120.6
C10—N2—Co1	126.9 (3)	C12—C13—C14	119.1 (4)
C6—N2—Co1	115.6 (3)	C12—C13—H13A	120.5
C15—N3—C11	117.5 (3)	C14—C13—H13A	120.5
C15—N3—Co1	131.7 (3)	C15—C14—C13	118.3 (4)
C11—N3—Co1	109.8 (2)	C15—C14—C17	121.9 (3)
N1—C1—C2	123.0 (4)	C13—C14—C17	119.9 (3)
N1—C1—H1C	118.5	N3—C15—C14	123.2 (3)
C2—C1—H1C	118.5	N3—C15—H15A	118.4
C3—C2—C1	118.1 (4)	C14—C15—H15A	118.4
C3—C2—H2A	120.9	O3—C16—O2	125.4 (4)
C1—C2—H2A	120.9	O3—C16—C11	117.3 (4)
C4—C3—C2	119.5 (4)	O2—C16—C11	117.3 (3)
C4—C3—H3A	120.2	O4—C17—O5	125.1 (4)
C2—C3—H3A	120.2	O4—C17—C14	117.8 (3)
C3—C4—C5	119.6 (4)	O5—C17—C14	117.1 (3)
C3—C4—H4A	120.2		

Symmetry codes: (i) −*x*+1, −*y*+1, −*z*+1.

### Hydrogen-bond geometry (Å, °)

**Table:**

Cg is the centroid of the *N*-pyridine ring.

**Table:**

*D*—H···*A*	*D*—H	H···*A*	*D*···*A*	*D*—H···*A*
O1—H1A···O3^ii^	0.84	1.82	2.629 (4)	159
O1—H1B···O6	0.88	1.94	2.755 (5)	154
O6—H6A···O4^iii^	0.85	2.40	3.232 (5)	166
O6—H6B···O4^iv^	0.83	2.01	2.829 (5)	170
C4—H4A···O2^v^	0.93	2.52	3.202 (5)	131
C7—H7A···O6^vi^	0.93	2.57	3.332 (6)	140
C8—H8A···O4^vii^	0.93	2.55	3.461 (6)	169
C9—H9A···O3^viii^	0.93	2.59	3.330 (6)	137
C12—H12A···O4^ix^	0.93	2.44	3.317 (5)	158
C15—H15A···O3^ii^	0.93	2.36	3.249 (5)	160
C2—H2A···Cg^iv^	0.93	2.72	3.547 (5)	150

Symmetry codes: (ii) *x*−1, *y*, *z*; (iii) −*x*, −*y*+1, −*z*+1; (iv) *x*, *y*+1, *z*; (v) −*x*+2, −*y*+2, −*z*+2; (vi) −*x*+1, −*y*+2, −*z*+2; (vii) −*x*+1, −*y*+1, −*z*+2; (viii) −*x*+2, −*y*+1, −*z*+2; (ix) *x*+1, *y*, *z*.
